# Image Analysis of 3D Conjunctival Melanoma Cell Cultures Following Electrochemotherapy

**DOI:** 10.3390/biomedicines8060158

**Published:** 2020-06-13

**Authors:** Miltiadis Fiorentzis, Periklis Katopodis, Helen Kalirai, Berthold Seitz, Arne Viestenz, Sarah E. Coupland

**Affiliations:** 1 Department of Ophthalmology, University Hospital Essen, 45147 Essen, Germany; 2 Biosciences, College of Health, Medicine and Life Sciences, Brunel University, London UB8 3PH, UK; katopodisper@gmail.com; 3 Liverpool Ocular Oncology Research Group, Department of Molecular and Clinical Cancer Medicine, Institute of Translational Medicine, University of Liverpool, Liverpool L69 3BX, UK; H.Kalirai@liverpool.ac.uk (H.K.); S.E.Coupland@liverpool.ac.uk (S.E.C.); 4 Liverpool Clinical Laboratories, Liverpool University Hospitals NHS Foundation Trust, Liverpool L69 3GA, UK; 5 Department of Ophthalmology, Saarland University Medical Center, 66424 Homburg, Germany; berthold.seitz@uks.eu; 6 Department of Ophthalmology, University Hospital Halle, 06112 Halle, Germany; arne.viestenz@uk-halle.de

**Keywords:** conjunctival melanoma, 3D cell culture, electroporation, electrochemotherapy, spheroid analysis, image analysis

## Abstract

Three-dimensional (3D) cell cultures represent small avascular tumors in vitro and simulate some of the biological characteristics of solid tumors, enhancing the evaluation of anticancer drug efficacy. Automated image analysis can be used for the assessment of tumor growth and documentation of changes in the size parameters of 3D tumor spheroids following anticancer treatments such as electrochemotherapy. The objective of this article is to assess the effect of various electroporation (EP) conditions (500–750 Volts/cm, 8–20 pulses, 100 µs pulse duration, 5 Hz repetition rate) combined with different bleomycin concentrations (1–2.5 ug/mL) on normal epithelial (HCjE-Gi) and conjunctival melanoma (CRMM1, CRMM2) 3D-cell cultures, through an automated image analysis and a comparison with standard histological assays. A reduction in tumor mass with loss of cell definition was observed after ECT (750 Volts/cm with eight pulses and 500 Volts/cm with 20 pulses) with bleomycin (1 μg/mL and 2.5 μg/mL) in the histological and immunohistochemical analyses of 3D CRMM1 and CRMM2 spheroids, whereas an increase in volume and a decrease in sphericity was documented in the automated image analysis and 3D visualization of both melanoma cell lines. For all other treatment conditions and for the HCjE-Gi cell line, no significant changes to their morphological features were observed. Image analysis with integrated software tools provides an accessible and comprehensive platform for the preliminary selection of homogenous spheroids and for the monitoring of drug efficacy, implementing the traditional screening methods.

## 1. Introduction

Cancer cells cultured in a two-dimensional (2D) environment offer a simple in vitro model to evaluate various chemical and biological compounds during investigation of novel candidate anticancer drugs [1,2,3,4]. Despite their cost-effectiveness and reproducibility, monolayers grown in culture flasks or petri dishes do not reproduce the development of normal or tumor tissues nor their complex spatial organization and interactions [5,6]. The development of three-dimensional (3D) in vitro models incorporates and imitates multiple elements of the tissue microenvironment (TME) and its organization, allowing for a more representative assessment of the biological performance of therapeutics in vivo [7,8]. 3D tumor spheroids are micro-sized cellular aggregates of tumor cells, which mimic various features of solid tumors, such as oxygen and metabolic gradients, and can be used as in vitro models for evaluation of the efficacy of anti-cancer therapeutic drugs before their testing in in vivo animal models or in clinical trials [9,10].

A reduction in tumor growth can be determined in 3D-spheroids by assessing the spheroid volume and perimeter, in order to analyze a particular drug’s potency [10]. Biochemical analysis of the spheroid growth, based on colorimetric, luminescence and fluorescence assays, requires optimization to ensure sufficient penetration of the assay reagents into spheroids [4,11]. Furthermore, inconsistencies can arise from multiple addition, aspiration and wash steps [4,11]. The manual measurement of length and width can be a time-consuming process, making large-scale analysis implausible [4,10]. Open-source automated image analysis software toolboxes, such as ImageJ, AnaSP, ReViSP are widely used for the assessment of various sized parameters of 3D tumor spheroids, and thereby tumor response at each step of a drug screen. Such software applications also allow the analysis of tumor growth after anticancer treatments, such as electrochemotherapy (ECT), with or without a cytostatic drug. The documented changes in the spheroid size parameters enable a more accurate approach regarding the applied electric field parameters as well as the drug concentrations [11].

Electrochemotherapy is an established treatment for skin and soft tissue metastatic lesions, deep-seated tumors of the liver, bone metastases and unresectable pancreas lesions [12,13,14,15,16]. Electroporation (EP) increases the permeability of the cell membrane after application of short electric pulses on both sides of the membrane, resulting in the formation of transient pores, allowing the delivery of large hydrophilic molecules to the cytosol [12,13,14,15,16]. ECT consists of the combination of a reversible EP and the therapeutic efficacy of chemotherapeutic agents, as a result of an increased membrane permeability, and the induction of cell death in the tumor cells of the targeted tissue [12]. ECT is an effective local, non-thermal treatment modality for the treatment of superficial and deep-seated nodules in progressive metastatic stages of different malignancies as well as for tumors not easily accessible (e.g., due to vessels or nerves in their proximity) or for tumors not amenable to excision or radiofrequency [13,15]. The aim of ECT treatment in such palliative settings is local tumor control and stabilization, an improvement in quality of life with symptom control, prevention of new local metastatic nodules, and potentially prolongation of survival [12]. The drawbacks of its application are localized pain, muscle contraction, erythema and edema [14]. The effect of ECT in ocular melanomas remains unclear, since the research and acquired knowledge in this field is very limited [17,18,19].

Conjunctival melanoma (CM) is a rare tumor with a heterogenous clinical presentation [20]. It is a subtype of mucosal melanoma that arises from atypical melanocytes in the basal layer of the conjunctival epithelium and is associated with a mortality of up to 30% [21,22,23]. It represents 2–5% of all eye tumors, 5–7% of melanomas in the ocular region and 0.25% of melanomas overall [23,24,25,26]. CM shows an incidence of 0.2–0.8 per million in the Caucasian population with rare cases reported in non-Caucasians and its incidence has risen in recent years, most likely due to increased UV exposure [27]. The mean age of presentation is ~65 years old [26,27]. CM can arise de novo or originate from primary-acquired melanosis (PAM; also termed conjunctival melanocytic intraepithelial neoplasia (C-MIN) and conjunctival nevi [28,29,30]. The rarity of CM has led to it being very difficult to conduct clinical trials regarding standardised therapy protocols for this disease [23]. The current standard of care is a wide excision of the tumor combined with a variety of adjuvant therapeutic modalities, such as topical chemotherapy with mitomycin, focal cryotherapy or brachytherapy and external beam radiotherapy, in order to prevent progression or recurrence [21,23]. ECT is integrated in the therapeutic approach of metastatic cutaneous melanoma, offering an alternative adjuvant option for non-excisable or multiple cutaneous and subcutaneous nodules [12]. Due to the similarities of CM to cutaneous and mucosal melanomas, the therapies effective in the treatment of these diseases may also show high potency in the management of CM and its metastatic lesions [27].

We present the application of ECT in CM 3D-spheroid models using various drug concentrations and electric field parameters. The aim of this paper is the analysis of morphological changes of the 3D spheroids during ECT application and the investigation of treatment efficacy not only based on biochemical assays and histological staining, but also on image analyses with documentation of size parameters, such as sphericity and volume per day of treatment. Such a detailed analysis has not been performed previously in CM 3D-spheroids, and these novel data provide a proposal for a supplementary tool in the investigation of the therapy efficacy, cytotoxicity and the structural behaviour of these cancer cell spheroids.

## 2. Materials and Methods

### 2.1. Cell Culture

Two conjunctival melanoma cells lines, CRMM1 and CRMM2, were kindly provided by Prof. Dr Martine Jager, Leiden University Medical Centre, the Netherlands [31]. The normal conjunctival epithelial cell line (HCjE-Gi), was kindly provided by Prof. Dr Colin Willoughby, University of Liverpool. All cell lines have been tested for mycoplasma and authenticated by Short Tandem Repeats (STR) before the experiments. CRMM1 and CRMM2 were grown in Ham’s F-12K (Kaighn’s) Medium containing 10% fetal bovine serum (Labtech International Ltd., East Sussex, UK) and 1% Penicillin Streptomycin (Life Technologies, Carlsbad, Kalifornien). The normal HCjE-Gi cell line was grown in Keratinocyte Medium with Human Recombinant Epidermal Growth Factor and Bovine Pituitary Extract (Life Technologies) containing 10% fetal bovine serum (Labtech International Ltd., Ringmer, Lewes BN8 5SY, UK). All cell lines were maintained as monolayers in 75 cm^2^ tissue culture flasks (Fisher Scientific, Loughborough, UK) at 37 °C in a humidified atmosphere containing 5% CO_2_.

### 2.2. D Cell Culture

CRMM1, CRMM2 and HCjE-Gi were cultured using Corning 96-well ultralow attachment plates (Sigma-Aldrich) according to the manufacturers’ instructions. Cells were seeded at various cell densities to verify the possibility of obtaining spheroids of at least 600–800 μm in diameter (0.5 × 10^3^, 1 × 10^3^, 3.5 × 10^3^, 5 × 10^3^, 7 × 10^3^, 10 × 10^3^ cells/well). The optimal density of spheroid formation and time of treatment was assessed based on the size, shape and hematoxylin and eosin (H&E) stain of each seeding density from day 3 to 15. Our previous data indicate a continuous growth of the spheroids (5 × 10^3^ cells) until day 3, a stable size until day 7, and a further growth on day 8 (REF). Spheroidal colonies grew on the bottom of the wells after about 3–5 days culture at 37 °C in atmosphere containing 5% CO_2_. As previously described, on day 3, the media was gently replaced and 200μL of fresh media was added in each well. Based on our previous data, days 3 and 8 were considered as the best timepoints for the spheroid treatment and the 5 × 10^3^ cells as the appropriate cell density [15]. Cells were seeded at 5 × 10^3^ cells/well to form spheroids at 37 °C in a humidified atmosphere containing 5% CO_2_. The culture medium was exchanged every other day. To remove culture medium, the end of a pipette tip was placed at the neck region of the well to avoid spheroid disruption. Each experiment was performed on three different dates, giving three technical replicates for each ECT condition.

### 2.3. Image Analysis

Growth of the 3D tumor spheroids/ellipsoids was monitored for 30 days, taking into account variations in spatial position, volume and shape. The image documentation of the 3D multicellular tumor spheroid growth was conducted daily. Phase-contrast imaging and morphological analyses of spheroids were carried out with a Nikon Eclipse TS100–TS100F Inverted Microscope (Carl Zeiss Microscopy GmbH, Jena, Germany) with 4×/1.0 detection, equipped with a Nikon D610 camera with 1.25-inch Extension Tube and T-mount adapter with T2 ring (Sony IMX-128-(L)-AQP CMOS sensor, square pixels of 35.9 × 24.0 mm length, 3872 × 3872 pixel resolution, sRGB color representation). The size parameters of 3D multicellular spheroids were measured using NIH ImageJ. The open-source Reconstruction and Visualization from a Single Projection (ReViSP) and ANAlyse SPheroids (AnaSP) software tools were used to achieve morphological 2D and 3D (volume, sphericity) parameters, and to select morphologically homogeneous spheroids, accordingly. In particular, for each spheroid the volume was calculated by using ReViSP on a single phase-contrast image. The detailed application of the volume-estimation method implemented in ReViSP was previously described [10]. 

The sphericity index (S) of each spheroid was calculated automatically by the AnaSP with the following equation

S = (π√(4A⁄π))/P(1)

where A is the Area and P is the Perimeter of each individual spheroid.

### 2.4. Spheroids Treatment with ECT

The spheroids were treated with two bleomycin concentrations (1 and 2.5 μg/mL) for 24- and 72 h on day 3 and day 8. Three EP conditions were applied using the voltage pulse generator (CliniporatorTM) designed by IgeaS.p.A. (Capri, Modena, Italy). The treatment conditions were as follows: (1) 0 μg/mL Bleomycin and no-EP; (2) eight square wave electric pulses (WEP) of 750 Volts/cm pulse strength; (3) 20 square WEP of 500 Volts/cm pulse strength; (4) 1 μg/mL Bleomycin with 8 square WEP of 750 Volts/cm pulse strength; (5) 1 μg/mL Bleomycin with 20 square WEP of 500 Volts/cm pulse strength; (6) 2.5 μg/mL Bleomycin with 8 square WEP of 750 Volts/cm pulse strength; (7) 2.5 μg/mL Bleomycin with 20 square WEP of 500 Volts/cm pulse strength. All EP treatments were conducted in the wells using two flat parallel stainless-steel electrodes with 100 μs pulse duration, 5 Hz repetition frequency on day 3 and 8 of spheroid culture. At 24- and 72-h following treatment, the spheroids were collected, fixed in 10% neutral buffered formalin (Leica Microsystems UK Ltd., Milton Keynes, UK) and prepared for histological and immunohistochemical (IHC) analysis.

### 2.5. Histological Analysis

Following treatment, the spheroids were collected in microcentrifuge tubes and 2% agarose was added to formalin-fixed with 4% PFA spheroids. The agar-embedded spheroids were transferred into tissue cassettes to be loaded onto the Bayer VIP E300 tissue processor. Tissue sections were stained with hematoxylin and eosin (H&E), according to standard protocols.

Immunohistochemistry was performed to detect the Ki-67 antigen using a mouse anti-human Ki-67 antibody (Clone MM1; Leica Microsystems UK Ltd., Milton Keynes, UK) at a dilution of 1:100 and a Dako EnVision Flex kit (Agilent Technologies LDA UK Limited, Cheadle, UK) according to the manufacturer’s instructions following dewaxing and heat-induced epitope retrieval in the Dako pretreatment module. The slides were incubated in a high-pH bath containing EnVisionTM FLEX target retrieval solution (Tris/EDTA buffer pH 9.0) at 96 °C for 20 min. EnVisionTM FLEX 3, -diaminobenzidine (DAB+) was used to visualize bound antibody. Sections were counterstained with Mayer’s hematoxylin (VWR International Ltd., Lutterworth, UK), blued with Scott’s tap water (Leica Microsystems UK Ltd., Milton Keynes, UK) and mounted with DPX mounting (Sigma, St. Louis, MO, USA). Human tonsil tissue was used as a positive control. Negative control sections were incubated with mouse IgG1. ImageJ2 (NIH Image, 2009) was used as a processing program for the multidimensional image analysis and for the Ki-67 proliferation evaluation.

### 2.6. Statistical Analysis

A Student’s *t*-test and two-way Anova were used to assess statistical significance of any changes observed in experiments. If data were not homoscedastic, an unpaired, two-tailed Student’s *t*-test with Welch’s correction was performed to account for variance. All statistical tests were performed using GraphPad Prism^®^ Software (Graphpad Software, Inc., San Diego, CA, USA). *p* values were denoted on graphs and interpreted as follows *p* = 0.01–0.05 = *; *p* = 0.001–0.009 = **; *p* <0.0001 = ***.

## 3. Results

### 3.1. Establishment of 3D Spheroid Assay for Treatment and Image Analysis

In order to investigate the ability of the CRMM1, CRMM2 and HCjE-Gi cell lines to form spheroids and the therapeutic and structural effect of ECT on the spheroids, we tested different cell concentrations as well as various electric field parameters and drug concentrations, using image analysis. For the validation and comparison of the image analysis outcome, additional histological and IHC assays were performed. An image documentation was conducted daily for 30 days. All the brightfield images were scanned and analyzed using ImageJ in combination with AnaSP and ReVisp software. All cell lines were examined as to whether they form spheroids when plated at 500 to 10.000 cells/well and for all the following experiments, the cell density of 5.000 cells was used. None of the melanoma cell lines formed tight spheroids, exhibiting more an assembly of loose aggregates, while the normal conjunctival epithelial cell line (HCjE-Gi) formed a tight compact aggregate, and they were spherical during all days and treatments (sphericity = 0.9). CRMM1 and CRMM2 formed spheroids in a variety of shapes with high diversity in their perimeter, diameter, sphericity and volume.

As shown in Table 1, the diameter of CRMM1 and CRMM2 from day 3 to day 11 (Day 9 with 72 h of treatment) of untreated spheroids, is increasing up to an average of 1239 and 1050 μm, respectively (Table 1). Based on these findings as well as the cell density and morphology of the spheroids via the image analysis, the appropriate ECT settings, drug type and drug concentrations were determined (Table 2).

### 3.2. Histology and Immunohistochemistry

**Figure 1:**
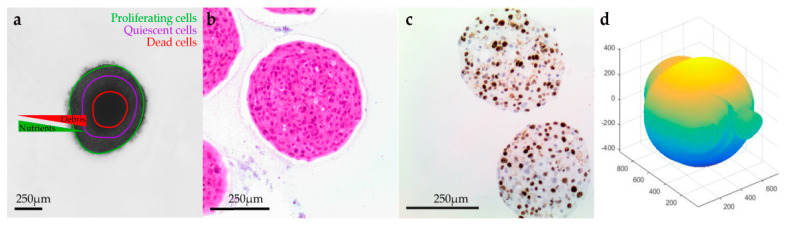
Shape and viability of the cells in a spheroid. (**a**) Brightfield image of HCjE-Gi spheroid grown in a 96-ULA. As presented, the cells that are lying in the periphery of the spheroid exchanging nutrients (O_2_, ATP) and debris (CO_2_) more effectively from the cells that are composing the necrotic center. (**b**) H&E where the necrotic center of dying cells is appearing in the center of the spheroid. (**c**) The external layer of the spheroid contains a lot of proliferating cells as shown by Ki-67 staining. (**d**) 3D reconstruction of the spheroid using ReViSP software.

H&E staining, together with the IHC analysis using Ki-67, indicated that the untreated spheroids during the experiments remained stable with a moderate proliferative rate. After the application of electric pulses, the growth of the melanoma spheroids varied, and their volume increased due to numerous necrotic cells, particularly at the centre, and detachments, usually at the edges. Spheroids treated with EP and bleomycin showed a higher sensitivity during the staining process, which resulted in a poor quality of cell retrieval for both H&E and Ki-67 staining. Only a small number of cells were Ki-67 positive in the spheroids’ periphery, while none were observed in the spheroid’s necrotic centre. All the spheroids presented a distinct dark pink necrotic centre in H&E staining, indicating an inability of these cells to exchange nutrients with the outer area of the spheroid (Figure 1).

**Figure 2:**
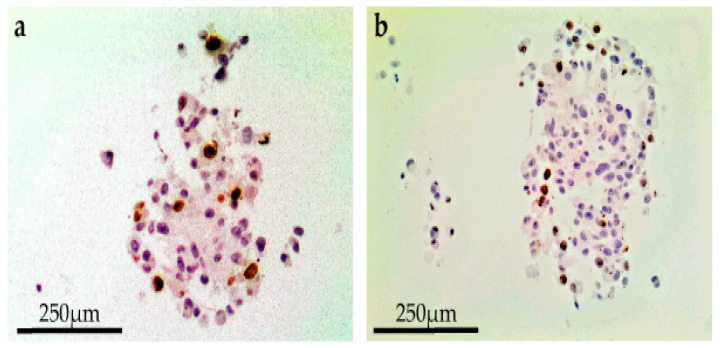
Immunohistochemistry with showing the nuclear staining of the Ki67 proliferative marker following ECT with 750 Volts/cm for 8 pulses combined with 1 µg/mL bleomycin shows reduction in the spheroid mass with necrotic center and confluent cells with multiple nuclei for CRMM1 (**a**) and CRMM2 (**b**) 24 h following treatment on day 8.

A reduced spheroid mass with necrosis of the core, loss of cell definition and formation of confluent cells with multiple nuclei was documented after combining 1 µg/mL bleomycin with EP at 750 Volts/cm for eight pulses. This effect was observed for all cell lines 24 h following treatment on day 8 (Figure 2). In terms of a decreased spheroid size and histological changes, this appeared to be most apparent in the CRMM2 cells following ECT.

### 3.3. Image Analysis following ECT with Bleomycin on the Spheroids

The three-dimensional surface and volume were visualized using ReViSP starting from a brightfield image. Furthermore, AnaSP was used to monitor different morphological parameters, including volume and sphericity index (S). Although the produced spheroid populations showed variable dimensions, we selected spheroids with a similar volume to guarantee the homogeneity of the 3D populations and to reduce bias to a minimum.

Up to day 3, the spheroids of normal conjunctival epithelial as well as melanoma conjunctival cell lines showed heterogeneity in both their volume and sphericity. The morphological and structural diversity increased after the application of EP alone or combined with bleomycin only in the melanoma cell lines. The process of daily documentation was continued 15 days after treatment with EP alone, drug alone or ECT. Due to detachment of peripheral necrotic cells or smaller secondary spheroids, significant shape changes were observed. In the analysis of spheroid volume, these detachments were included in the measurement, hence the volume of the spheroid increased in the digital image analysis. 

**Figure 3:**
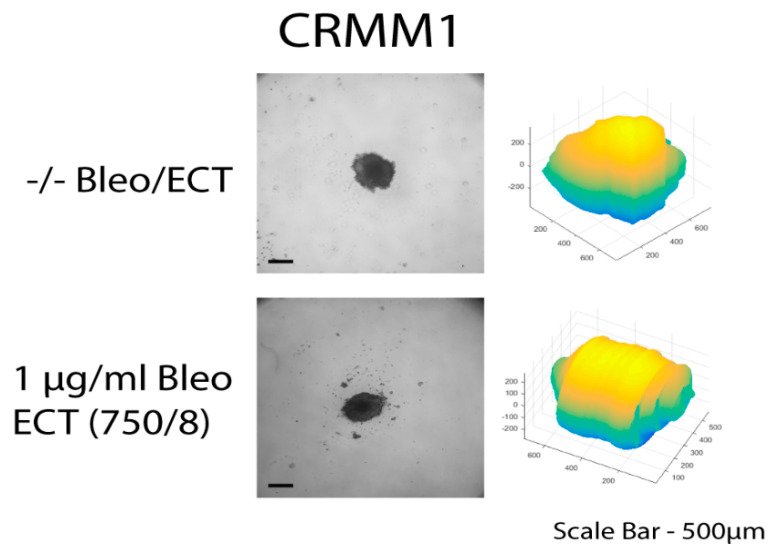
CRMM1 cell line spheroids on day 8 without treatment as well as following ECT (750 V/8 p) combined with 1 µg/mL bleomycin. Brightfield image and 3D volume reconstruction showing the change in size parameters after treatment. Further analysis of the cell line spheroids across the days and the types of treatment including H&E staining and 3D reconstructions are provided in Supplementary Figure S1.

**Figure 4:**
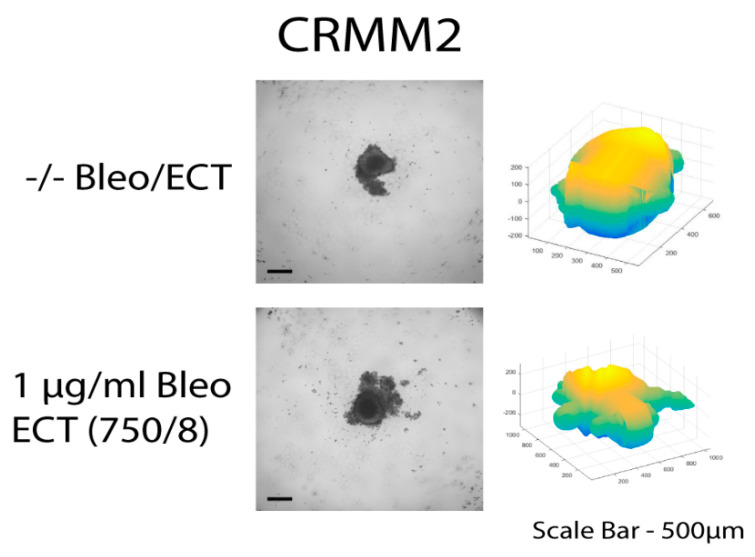
CRMM2 cell line spheroids on day 8 without treatment as well as following ECT (750 V/8 p) combined with 1 µg/mL bleomycin. Brightfield image and 3D volume reconstruction showing the change in size parameters after treatment. Further analysis of the cell line spheroids across the days and the types of treatment including H&E staining and 3D reconstructions are provided in Supplementary Figure S1.

The untreated spheroids and the spheroids after bleomycin alone showed no significant changes in their periphery. After ECT with bleomycin, the edges and the cells exhibited low adhesion and density, resulting in a detachment from the main spheroid body. The peripheral ‘peeling’ of the cells continued over subsequent days, and non-attached or weakly attached cells were removed during the staining progress. The loss of spheroidal cohesion is presented in Figure 3 and Figure 4 as well as in the H&E staining in the Supplementary Figures S1 and S2. Treated spheroids could not be retrieved unharmed and only sections of the main spheroid body could be stained. The effect was more prominent in CRMM1 and CRMM2 cell lines 72 h after treatment on day 3 with ECT (500 V/20 p) combined with 1 and 2.5 µg/mL bleomycin and ECT (750 V/8 p) combined with 2.5 µg/mL bleomycin. A similar result was observed 24 h after ECT (750 V/8 p) on day 8 combined with 1 µg/mL bleomycin for both melanoma cell lines (Figure 3 and Figure 4).

**Figure 5:**
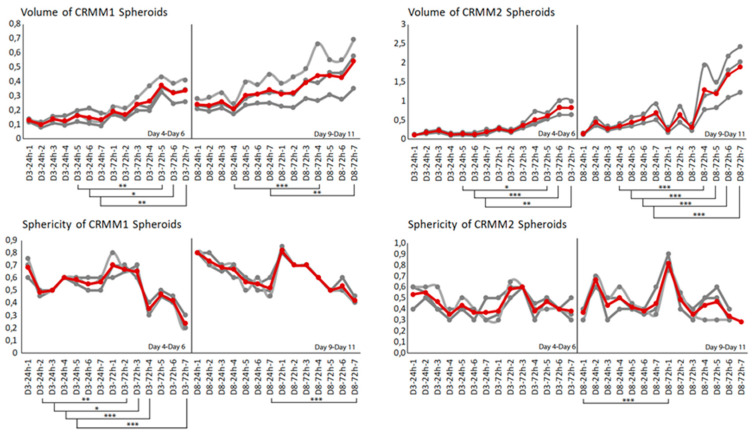
Mean of three volume and circularity measurements for CRMM1 and CRMM2 across the days and treatments. As shown, as the pulses and Voltages increase along with the days of treatment, the volume and the sphericity of the spheroids vary. The grey lines represent the triplicates of the experiment; the red line is the mean curve. The volume is increasing during the treatments and time while the spheroids lose their circularity as a result of cytotoxicity of the drug that has penetrated the spheroid periphery.

**Figure 6:**
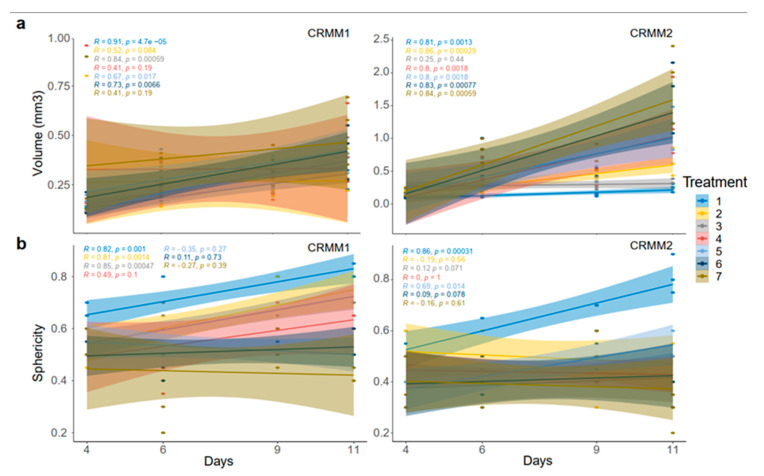
Volume (**a**) and sphericity (**b**) curves across the days of treatment for both CRMM1 and CRMM2. As shown, the non-treated spheroids (blue line) remain the same in the volume graphs while the sphericity of the spheroids increased with time. On the other hand, treated spheroids, especially in the condition 5, 6 and 7 with the EP+ bleomycin, the volume increased within the treatment period and the spheroids lost their circularity.

With respect to the analysis of quantitative features of the spheroids after ECT with cytostatic agents, the sphericity of the treated spheroids was also assessed. The variability in sphericity was partially lost for the untreated spheroids for both cell lines acquiring a more spherical shape and reaching a circularity greater than or close to 0.9 after 11 days of culture in low-attachment plates, as depicted with a peak in the sphericity diagram (Figure 5). More prominent morphological changes caused by cell detachment or peeling off one or more small secondary spheroids were more frequently documented in spheroids with a less spherical, more spindle-like morphology, either before therapy initiation or due to changes appearing rapidly after treatment. This observation is more apparent for the conditions 4 and 7, 72 h after treatment on day 3 as well as 24- and 72 h after day 8 in the volume and sphericity diagrams, where CRMM1 and CRMM2 spheroids show a lower SI and a greater volume (Figure 5 and Figure 6). The heterogeneity of the spheroid shape following ECT was associated with alterations in the dimension of the inner core presenting necrotic areas and in the thickness of the surrounding outer area consisting of proliferative cells.

## 4. Discussion

The 3D-cell cultures exhibit complex cell–cell and cell–extracellular matrix interactions and possess chemical gradients (oxygen, nutrients and metabolites), better reflecting the in vivo behavior of tumor cells [32,33]. Methods of culturing cells in 3D include polarized cultures using trans-well inserts, multicellular spheroids, bioreactors, matrix embedded cells, scaffold-based systems, hollow-fiber bioreactors and organotypic slices [7,32,34]. Multicellular tumor spheroids depict a reproduction of small avascular tumors in vitro [35,36]. Despite the advantages of spheroids and their establishment in cancer research, monolayer cell cultures remain the method of choice for many anticancer drug screens [32]. Factors such as slow growth, heterogeneity in spheroid shape and their growth curves, as well as the difficulties associated with an accurate determination of the viability, could all render the culturing process too strenuous and imperil the reliability of data. Therefore, a 3D visualization, combined with the automatized calculation of morphological parameters, could enrich and facilitate the monitoring process for the selection of homogenous-sized spheroids before treatment initiation, to assess the efficacy and cytotoxicity of novel therapeutics.

The purpose of our image analysis was to compare the effects of ECT with bleomycin on conjunctival epithelial and tumor cell lines using 3D models with the standard histological screening assays. An image analysis and the integrated algorithms offer a quantitative characterization and a more precise calculation of various morphological features of the tested spheroids. Moreover, an image analysis complements the established assays for the spheroid monitoring, while it provides a comprehensive platform with a variety of techniques, such as statistical analysis, graph representation and computational topology and, therefore, 3D visualization [17]. In previous studies of our group, various EP parameters and differing concentrations of 5-FU, Mitomycin C and bleomycin were tested to identify the structural changes of the spheroids histologically and chemically, and to assess the toxicity of the tested drugs on conjunctival 2D and 3D cell models [17]. The CRMM1 cell lines were most sensitive to 5-FU and the CRMM2 cell lines to MMC but the cytotoxic effect of the drugs was not further enhanced by EP [17]. Bleomycin was subsequently selected as the drug of choice to investigate the potency of ECT on conjunctival tumor spheroids as a novel therapeutic candidate. ECT could present a less invasive therapeutic option, especially for larger tumors, offering the advantage of avoiding the complications and adverse effects of chemotherapy through the application of lower applied doses.

In the present study, the applied image analysis detected a heterogeneity in the morphology of the examined spheroids in terms of shape and volume for the normal epithelial and conjunctival melanoma cell lines before treatment initiation, as well as significant changes in the parameters after the application of different therapeutic settings with EP or ECT. Other groups reported on the importance of monitoring different morphological criteria, such as diameter, perimeter, area, volume and sphericity, the variability of which may affect the reproducibility of the results obtained [37,38,39,40,41]. This observation especially applies in the investigation of multiple therapeutic conditions and constitutes an aid to preselect spheroids with similar characteristics via image analysis, ensuring the reliability of the results as well as homogenous populations accessible for automated high-throughput screens and data mining. The correlation between an irregular shape or a lower S and a cell detachment or growth of one or more secondary spheroids was presented in our image analysis. Spheroids homogenous in volume can vary in shape because the irregular morphology of the three-dimensional cultures can affect the number of cells exposed to high levels of nutrients, oxygen and xenobiotics and, consequently, the percentage of actively proliferating cells [42].

Although a preliminary evaluation of the spheroid morphology following ECT through the histological images shows a reduction of size 24 h after day 3 and 8, the determination of the metabolic activity remains essential. In contrast to this finding, an increase in volume was documented through the three-dimensional visualization and image analysis using automated software tools. The automated volume reading can be influenced by cell detachment or the formation of one or more secondary spheroids in the periphery, but also by changes in the metabolic activity, the Ki-67 proliferation rate, the proportion of necrotic cells and the size of the necrotic core [32]. The previous data of our group on conjunctival melanoma cell lines after ECT with bleomycin showed a decreased Ki-67 growth fraction in all three cell lines on day 3 as well as on day 8 compared to the findings without treatment. The combined treatment led to an 89.5% reduction in the proliferation of the CRMM1 and to an ~88% reduction in the CRMM2 on day 3. Bleomycin treatment alone showed an increase in the Ki-67 staining by 61.6% of the CRMM1 cells and 59.5% of the CRMM2 cells [17]. The spheroid volume is dependent on the cell density distribution and the concentric layering profile. The size of the core region must not appear proportional to the size of the spheroid, and the thickness of the outer area is associated with the nutrient and oxygen uptake, varying upon the spheroid size [43].

The presented data of image analysis represent a supplementary tool in the investigation of the therapy efficacy, cytotoxicity and the spheroid structural behaviour following ECT with bleomycin in conjunctival melanoma 3D spheroid cultures. This approach will enable the implementation of standard biochemical and histological analyses and could also offer a simpler and quicker assessment for a preliminary selection of homogenous cancer cell spheroids and the optimization of the experimental design, improving on the reliability of the results. Further refinement of algorithms from various computational fields could be integrated in the traditional image analysis to incorporate quantitative measurements of the single cell, and therefore improve the accuracy of data, particularly regarding the efficacy of ECT as a potential anticancer treatment for ocular melanoma.

## Figures and Tables

**Table 1:** The average values for CRMM1 and CRMM2 of untreated spheroids across the days.

CRMM1 (*n* = 3) Days	Perimeter (μm)	Circularity	Diameter (μm)	Volume (mm^3^)
D3	2724	0.68 (±0.08)	877	0.193 (±0.04)
D4	3146	0.65 (±0.05)	898	0.129 (±0.01)
D6	3168	0.7 (±0.10)	981	0.19 (±0.02)
D8	2025	0.83 (±0.06)	648	0.239 (±0.03)
D9	2642	0.8 (±0.0)	866	0.307 (±0.05)
D11	3725	0.82 (±0.03)	1239	0.312 (±0.08)
**CRMM2 (*n* = 3)**				
D3	3244	0.53 (±0.12)	906	0.176 (±0.10)
D4	2600	0.55 (±0.05)	935	0.112 (±0.01)
D6	2680	0.58 (±0.08)	853	0.270 (±0.03)
D8	1973	0.99 (±0.03)	689	0.170 (±0.04)
D9	2206	0.67 (±0.06)	722	0.135 (±0.21)
D11	2043	0.82 (±0.08)	1050	0.247 (±0.06)

**Table 2:** Spheroid treatment conditions and their labels for the following analysis.

Label	Condition
1	NT
2	750 V/8 p
3	500 V/20 p
4	1 μg–750 V/8 p
5	1 μg–500 V/20 p
6	2.5 μg–750 V/8 p
7	2.5 μg–500 V/20 p

## Supplementary Materials

Supplementary materials can be found at https://www.mdpi.com/2227-9059/8/6/158/s1.
