# Supplementary material for: Image Analysis of 3D Conjunctival Melanoma Cell Cultures Following Electrochemotherapy
Source: Biomedicines. 2020 Jun 13;8(6):158. doi: 10.3390/biomedicines8060158 (PMC7344416; doi:10.3390/biomedicines8060158)

**Supplementary Figure S1.** CRMM1 cell line spheroids across the days and the types of treatment. H&E staining and 3D reconstructions were conducted to examine the changes on the shape spheroid and the necrotic center.


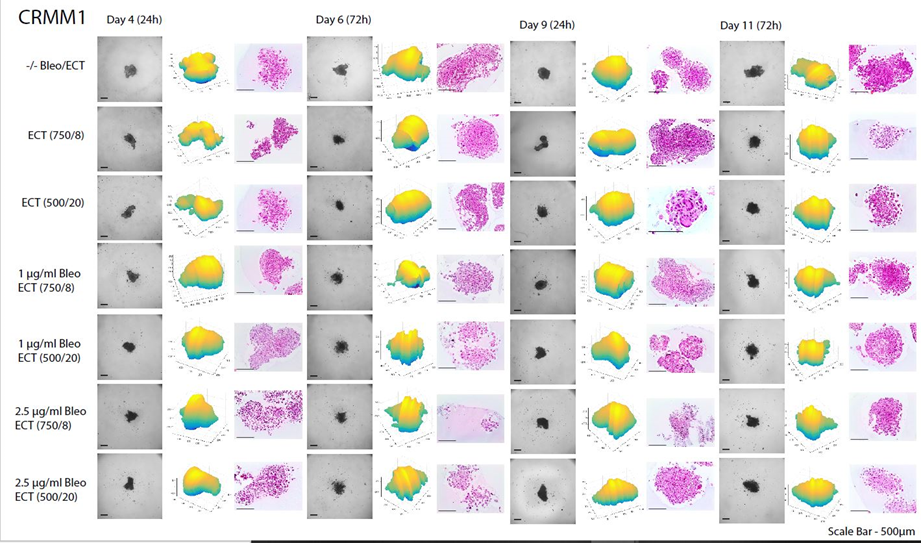

Supplement: Supplementary file 1 [file biomedicines-08-00158-s001.zip › biomedicines-799527-supplementary/Supplementary Figure S1_CRMM1.docx]
